# Behavior of Zinc Incorporation into Fuel Deposits in Borated and Lithiated Water with Dissolved Zinc

**DOI:** 10.3390/ma13194317

**Published:** 2020-09-28

**Authors:** Do Haeng Hur, Kyeong-Su Kim, Hee-Sang Shim, Jinsoo Choi, Kyu Min Song

**Affiliations:** 1 Korea Atomic Energy Research Institute, 989-111, Deadeok-daero, Yuseong-gu, Daejeon 34057, Korea; zmsqkdnl97@naver.com (K.-S.K.); hshim@kaeri.re.kr (H.-S.S.); 2 Central Research Institute of Korea Hydro & Nuclear Power Co., Ltd., 1312-70, Yuseong-daero, Yuseong-gu, Daejeon 34101, Korea; jinsoochoi@khnp.com (J.C.); kmsong001@khnp.com (K.M.S.)

**Keywords:** fuel deposit, zinc addition, zinc incorporation, site preference energy, radiation field

## Abstract

The objective of this study was to investigate the behavior of zinc incorporation into newly forming fuel deposits and pre-formed deposits in a simulated pressurized water reactor coolant including 1000 ppm of boron and 2 ppm of lithium at 328 °C. Zinc was incorporated into fuel deposits that were being newly nucleated and grown on nuclear fuel cladding tubes in a zinc-containing coolant. The zinc incorporation resulted in a decrease in the lattice constant of the deposits, which was attributed to the decrease in larger iron content and the corresponding incorporation of smaller zinc in the deposits. However, zinc incorporation was not found, even after the fuel deposits pre-formed before zinc addition were subsequently exposed to the 60 ppb of zinc coolant for 500 h.

## 1. Introduction

Zinc addition into a reactor coolant system (RCS) was first applied at Hope Creek Unit for a boiling water reactor (BWR) in 1987 and Farley Unit 2 for a pressurized water reactor (PWR) in 1994 [1,2]. The original purpose of the zinc addition was to suppress the buildup of radiation fields, and since then, plant experience has demonstrated that zinc addition significantly decreases dose rates. The major source of the radiation fields in PWRs is ^60^Co and ^58^Co isotopes, which are formed in the reactor core by the neutron capturing of ^59^Co and by ^58^Ni (n,p) reaction with fast neutrons, respectively [3]. The decrease in radiation fields from zinc addition is ascribed to a reduction of the corrosion release rates for ^59^Co and ^58^Ni from the surfaces of RCS materials [4,5,6], especially Ni-based Alloy 600 or 690 steam-generator tubes. Zinc acetate has been added to the RCS coolant, which is soluble in water.

Zn^2+^ ions have been reported to replace Ni, Fe, Co, or radioactive Co ions in spinel-type oxides formed on nickel-based alloys and stainless steels under RCS conditions [7,8,9,10]. Because the zinc-incorporated oxides are thermodynamically more stable and protective [5,11,12], the corrosion and corrosion release rates of the RCS materials are significantly mitigated, resulting in a reduction of the radiation source term.

A beneficial effect of zinc was also reported in mitigating the stress corrosion cracking (SCC) [13,14,15] and corrosion fatigue [16] of Ni-based alloys and stainless steels in simulated RCS coolant environments of PWRs. Accordingly, as of 2014, there were 85 PWRs worldwide, and most BWRs have implemented the zinc injection technology into the RCS [17]. As mentioned above, a lot of research on zinc addition has been performed from the viewpoint of the corrosion rate [5,6,18,19], corrosion release rate [4,5,6,10,20], oxidation kinetics [7,12,21,22,23], dose rate [20,24,25,26], and zinc incorporation [5,6,7,8,9,10,11,12,20] in the oxide films of Ni-based alloys and stainless steels in laboratories and operating PWRs. However, there is relatively little open literature on the effects of zinc injection on fuel deposits, possibly because of experimental difficulty in laboratories and extremely high-level radiation from burned fuel assemblies. These studies are related to plant experience [27,28,29,30,31,32,33,34] and changes in the composition [28,29,30], magnetic properties [27], morphology [34], and deposition mass [34,35,36,37] of fuel deposits caused by zinc addition.

Despite its advantages, there are still concerns limiting the zinc addition initiative, especially in high-duty cores. The major concerns are related with the potential impacts on fuel integrity. First of all, added zinc could induce the buildup of corrosion products on fuel cladding surfaces [34,35,36,37] or the precipitation of zinc oxide and zinc-containing silicates within fuel deposits [32,38]. The zinc in the coolant could be incorporated into the fuel deposits [27,28,29,30] and substitute cations, as in the oxide films of Ni-based alloys and stainless steels. The zinc could also affect the corrosion rate of fuel claddings [39,40]. In previous work [37], the fuel deposition amount at 100 ppb of zinc increased by approximately 55%, compared with that at 0 ppb of zinc. The deposition behavior was also elaborated using the electrostatic force between the deposited particles and fuel-tube surface. As described above, the beneficial effect of zinc on dose-rate reduction has been investigated in connection with the corrosion of the RCS materials. Therefore, in this study, the behavior of zinc incorporation into newly forming fuel deposits and pre-existing fuel deposits was investigated under simulated RCS coolant conditions of PWRs using a fuel-deposit deposition system. The zinc incorporation and the resultant reduction effects on radiation fields are extensively discussed.

## 2. Experimental Methods

### 2.1. Preparation of Test Specimens and Solutions

Zirconium-based nuclear fuel cladding tubes were used for the fuel-deposit deposition and zinc-incorporation tests. The chemical composition together with the mechanical properties is given in Table 1. The dimension of each cladding tube specimen was 0.95 cm in outer diameter, 0.057 cm in wall thickness, and 55 cm long. One side of each specimen was leak-tightly welded with a zirconium plug. A cartridge heater was installed inside the tube specimen and used as a heat source. Before tests, the outer surface of the tube was ultrasonically cleaned in acetone and deionized water in sequence.

The test solution used was composed of 1000 ppm of boron as boric acid (H_3_BO_3_) and 2 ppm of lithium as lithium hydroxide (LiOH) in demineralized water to simulate the RCS coolant of PWRs. If necessary, depleted zinc acetate dihydrate (Zn(C_2_H_3_O_2_)_2_·2H_2_O) was added to the boron/lithium solution at 20 or 60 ppb of zinc. The solution was prepared inside two RCS coolant containers with a volume of 200 L.

The deposit source (precursor) solution for the deposition tests was made up by adding nickel- and iron-ethylene diamine tetra-acetic acid to the simulated RCS coolant including 1000 ppm of boron and 2 ppm of lithium. The concentration of the deposit source was dependent on the test conditions, and the final source solution was put into a deposit source container.

### 2.2. Test for Zinc Incorporation into Newly Forming Fuel Deposits

**Figure 1:**
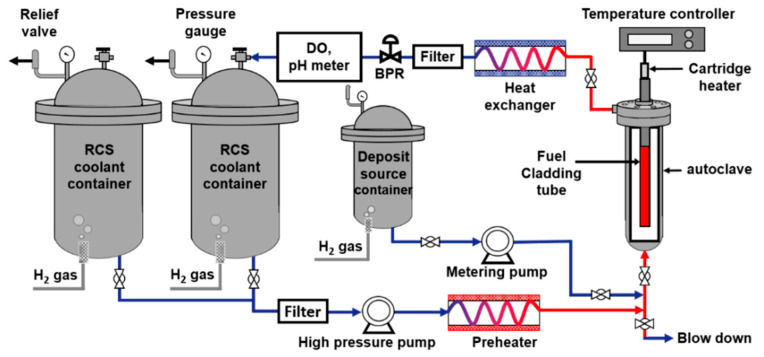
The coolant circulation loop system for fuel-deposit deposition and zinc incorporation.

Zinc-incorporation behavior during deposit formation on fresh cladding tubes was investigated using a coolant circulation loop system for fuel-deposit deposition. As shown in Figure 1, the system mainly consisted of an RCS coolant circulation part, a test part, and a deposit source addition part. The prepared cladding tube specimen was vertically installed in an autoclave of the test part.

The oxygen content dissolved (DO) in all the solutions was controlled to be less than 5 ppb by continuously blowing hydrogen gas (99.999% purity) inside the solution containers. The hydrogen content dissolved (DH) in the solutions was adjusted to 35 cm^3^/kg H_2_O (STP) by pressurizing the solution containers with hydrogen gas. The simulated RCS coolant was circulated with a flow rate of 280 mL/min through a high-pressure pump, a pre-heater, an autoclave, and a heat exchanger. The RCS coolant entering the autoclave was pre-heated to 325 °C, and the temperature of the coolant running through the annular space around the tube specimen was finally kept at 328 °C. The system pressure of the autoclave was regulated to 130 bar with a back pressure regulator (BPR). The heat flux supplied to the tube surface by the cartridge heater was 65 W/cm^2^.

After these parameters reached target conditions, the injection of the deposit source solution into the downward flow of the pre-heater was started using a micro-metering pump at an injection rate of 1.0 mL/min. Therefore, the injected deposit source solution was blended with and diluted in the RCS coolant flow, which finally gave a concentration of 500 ppb of iron and 280 ppb of nickel without zinc (Case #1) and with 20 ppb of zinc (Case #2) in the autoclave. The duration of each deposition test was 600 h. The experimental conditions for the tests are summarized in Table 2.

### 2.3. Test for Zinc Incorporation into Pre-Existing Deposits

The behavior of zinc incorporation into pre-existing deposits was investigated using cladding tubes with pre-formed thick deposits. To prepare the pre-existing deposits, deposit deposition on a cladding tube was first performed in the simulated RCS coolant including 1000 ppm of boron and 2 ppm of lithium without zinc at 328 °C using the coolant circulation loop system shown in Figure 1. In this test, the concentration of the deposit source solution containing nickel and iron increased to form a thick deposit layer. The injected source solution was blended with the simulated RCS coolant flow, resulting in a concentration of 8 ppm of iron and 6 ppm of nickel in the autoclave. After deposition for 240 h, the loop system was shut down and the cladding tube covered with deposits was removed from the autoclave to analyze the deposits (Case #3). In Case #4, after the same deposition for 240 h as in Case #3, the loop system was shut down and cleaned by circulating the simulated RCS coolant comprising 1000 ppm of boron and 2 ppm of lithium to remove the residual deposit source in the loop. Then, the loop system was restarted and reached the steady state, and the deposited cladding tube was exposed to the circulating RCS coolant including 1000 ppm of boron and 2 ppm of lithium with 60 ppb of zinc at 328 °C for 500 h without deposit source injection. The detailed test conditions are given in Table 3.

### 2.4. Fuel Deposit Analysis

After each test was finished, the cladding tube specimen was cut into segments to characterize the fuel deposits microscopically. The morphology of the deposits was examined by utilizing a scanning electron microscope (SEM). Fuel deposits were collected with a plastic knife from the deposited tube surfaces and analyzed through X-ray diffraction (XRD) examinations. XRD spectra were obtained in the 2θ range of 20° to 80° with a scan rate of 1°/min using a high-resolution diffractometer with copper K*α* radiation operating at 40 kV and 0.3 A. Transmission electron microscopy (TEM) specimens were made by machining fuel deposits using a focused ion beam (FIB) technique. Scanning TEM (STEM) micrographs of fuel deposits were obtained using a transmission electron microscope operated at 200 kV. The chemical composition of the deposits was examined using energy dispersive X-ray spectroscopy (EDS) apparatus installed on the TEM.

The two-dimensional (2D) porosities of the fuel deposits were measured using SEM micrographs, which were obtained from the cross-sections of the FIB-milled trenches of the deposits. Image analysis software, ImageJ, was used to determine the 2D porosity values from the SEM photographs.

## 3. Results and Discussion

### 3.1. Behavior of Zinc Incorporation into Newly Forming Fuel Deposits

**Figure 2:**
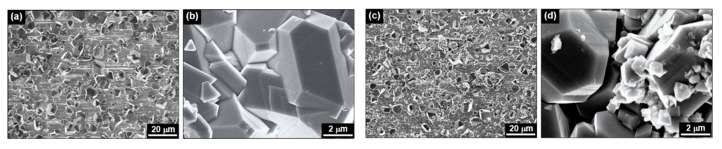
SEM micrographs of fuel deposits formed (**a**,**b**) under the no-zinc condition (Case #1) and (**c**,**d**) at the 20 ppb zinc concentration (Case #2).

Figure 2 shows SEM photographs of the fuel deposits formed on the surfaces of the fuel cladding tubes in the coolant without (Case #1) and with 20 ppb of zinc (Case #2). Regardless of zinc addition, deposit particles of polyhedral shape were evenly formed on the fuel cladding tube surfaces. The magnified particles showed clear crystalline facets and ranged up to several microns in size. It seems that submicron particles were observed at 20 ppb of zinc more frequently than at 0 ppb of zinc.

**Figure 3:**
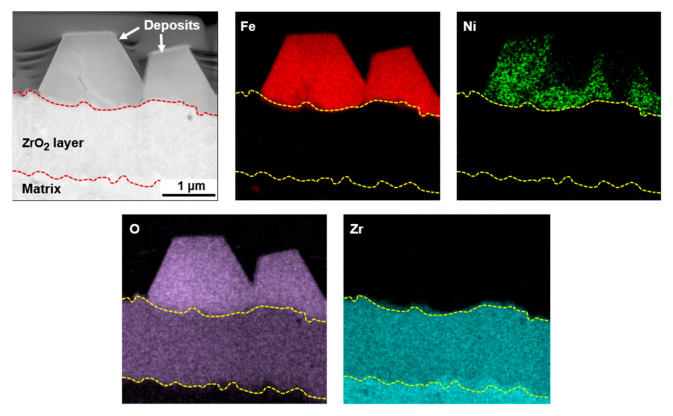
Scanning TEM (STEM) micrograph and elemental EDS maps of fuel deposits formed in the coolant without zinc (Case #1).

The deposited particles were vertically machined to make TEM specimens using the FIB milling technique and examined using STEM-EDS. Figure 3 presents a STEM micrograph and EDS elemental composition maps of deposit particles formed in the coolant without zinc (Case #1). Regardless of the particle size, Fe, Ni, and O were detected. Therefore, the elemental mapping indicates that the polyhedral-shaped particles are oxides consisting of Fe and Ni. From the STEM-EDS results of the deposit particles, the deposits were identified as nickel ferrites with a spinel stoichiometry of Ni_0.26_Fe_2.74_O_4_ on average. On the other hand, a zirconium oxide layer with a thickness of 1~2 µm was observed, which was formed through the internal oxidation of the zirconium-based fuel cladding tube during the test.

**Figure 4:**
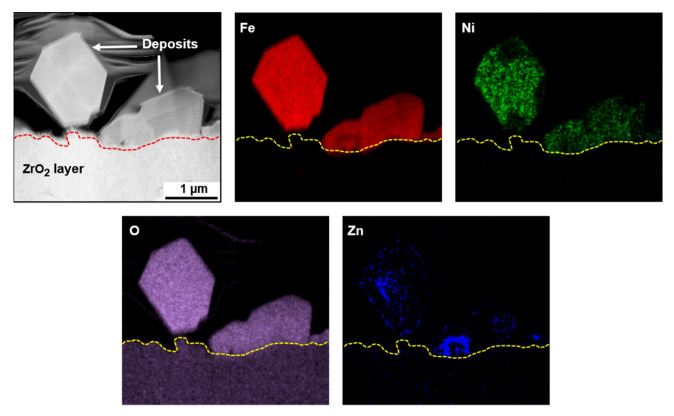
STEM micrograph and elemental EDS maps of fuel deposits formed in the coolant with 20 ppb of zinc (Case #2).

Figure 4 shows a STEM micrograph and elemental EDS maps of the fuel deposits formed in the coolant with 20 ppb of zinc (Case #2). As seen in the maps, zinc was found in the particles and the zinc content detected in the deposits ranged up to 3.2 at %. From the quantitative STEM-EDS analyses, the deposits were characterized as nickel ferrites containing zinc, with an average spinel formula of Ni_0.23_Zn_0.14_Fe_2.63_O_4_. On the other hand, zinc was not found in the zirconium oxide layer, indicating that zinc did not react with zirconium oxide.

**Figure 5:**
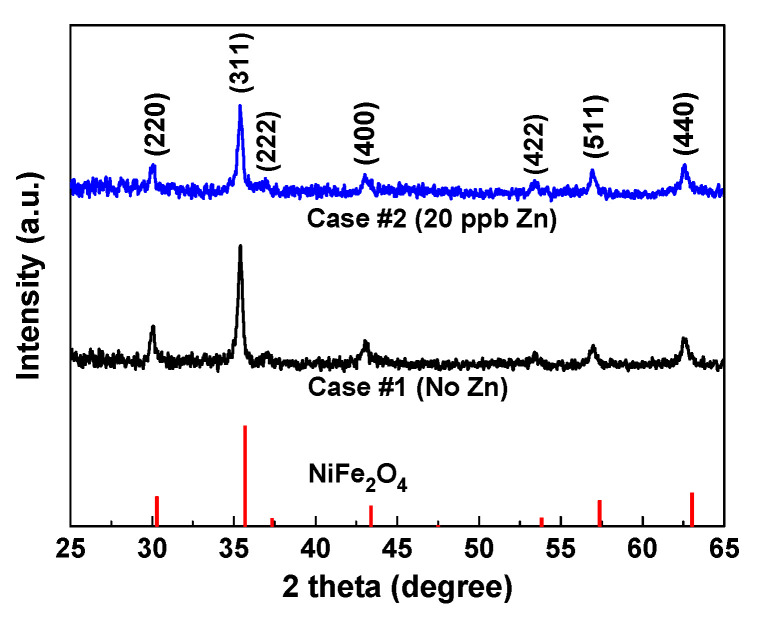
XRD spectra from fuel deposits formed under the conditions of Case #1 and Case #2.

The XRD spectra and resultant Miller indices obtained from the deposits formed under the no-zinc condition (Case #1) and at 20 ppb of zinc (Case #2) are shown in Figure 5. The Miller indices for each diffraction were assigned using the relation of the Bragg law combined with the equation for the distance between adjacent planes for the crystal system [41]. All the Bragg planes indexed in the figure corresponded to the cubic spinel structure in the Fd-3m space group. The most intensive diffraction was recorded from the (311) plane, and other distinct diffractions were not detected from the XRD spectra. As shown in Figure 5, all the diffractions largely coincided with the reference XRD spectra of NiFe_2_O_4_, having the cubic spinel structure (PDF 54-0964). The slight shift of the characteristic diffraction angles is due to the difference in the chemical compositions between the deposits and the reference NiFe_2_O_4_. Therefore, the XRD analysis together with the STEM-EDS data lead to the conclusion that the fuel deposits are cubic crystalline spinel oxides

The lattice constants for the cubic fuel deposits can now be calculated using the following equation [41]:

$$a=\frac{\lambda \sqrt{h^{2}+k^{2}+l^{2}}}{2sin\theta }$$

where *a* is the lattice constant, *λ* is the wavelength of the incident beam (1.54 Å for the K*α* radiation of copper), (*h*, *k*, *l*) are the Miller indices of the diffraction plane, and *θ* is the diffraction angle.

Since the systematic error in the lattice constant decreases as the Bragg angle increases, the value of the lattice constant was measured at the highest diffraction angle of the XRD spectra (2*θ* = 62.50°). The lattice constant of the fuel deposits formed under the no-zinc condition was 8.396 Å. The lattice constant of spinel NiFe_2_O_4_ has been reported to be in the range of 8.33~8.35 Å [42,43,44,45]. Therefore, the lattice constant of the deposits is somewhat larger than that of NiFe_2_O_4_. The effective radii of important metallic cations increase in the following order [46]: 0.69 Å for Ni^2+^ < 0.74 Å for Zn^2+^ < 0.78 Å for Fe^2+^. Comparing the deposits (Ni_0.26_Fe_2.74_O_4_) with the NiFe_2_O_4_, it is evident that the deposits contain the larger Fe^2+^ cations more and the smaller Ni^2+^ cations less. Consequently, the increase in the lattice constant of the deposits formed without zinc is due to the concentration of the bigger Fe^2+^ cations and the resultant deficiency of the smaller Ni^2+^ cations. Meanwhile, the lattice constant of the fuel deposits formed at 20 ppb of zinc was determined to be 8.385 Å. That is, the lattice constant was decreased from 8.396 to 8.385 Å by the addition of 20 ppb of zinc. Note that the chemical composition of the deposits changed from Ni_0.26_Fe_2.74_O_4_ at zero zinc to Ni_0.23_Zn_0.14_Fe_2.63_O_4_ at 20 ppb of zinc. Therefore, the decreased lattice constant of the deposits formed at 20 ppb of zinc is due to the decrease in the bigger Fe^2+^ cations and the corresponding incorporation of the smaller Zn^2+^ cations.

### 3.2. Behavior of Zinc Incorporation into Pre-Existing Fuel Deposits

**Figure 6:**
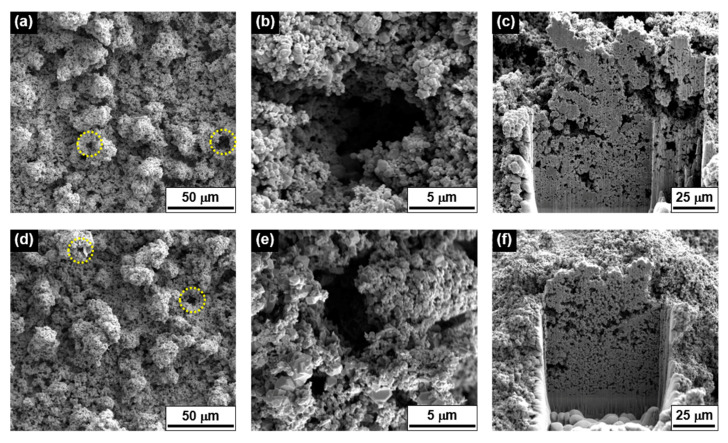
SEM micrographs of fuel deposits formed (**a**–**c**) under the condition of Case #3, and (**d**–**f**) after the subsequent exposure of the deposits formed under the condition of Case #3 to the 60 ppb of zinc coolant under the condition of Case #4. (**b**,**e**) show examples of large boiling chimneys. (**c**,**f**) are the cross-sections of the deposits machined using the focused ion beam (FIB) technique.

Figure 6 shows SEM micrographs of the surfaces and cross-sections of the thick fuel deposits formed under the no-zinc condition of Case #3 and after the subsequent exposure to the 60 ppb of zinc coolant under the condition of Case #4. From the top-down view of the deposit surfaces, it is observed that the deposits have numerous micro-pores and uneven thickness over the cladding surfaces. The dotted circles in Figure 6a,d show examples of large micro-pores. It can also be seen that the deposits are composed of particles with various sizes. A distinct morphological change was not found after the pre-formed deposits were exposed to the 60 ppb of zinc coolant for 500 h. Micro-pores were clearly observed on the cross-sections of the fuel deposits as shown in Figure 6c,f, which were milled using the FIB. These pores are formed when the coolant boils on the heated tube surface and the vapor bubbles depart from the heated surface through the deposits to the bulk, which are called boiling chimneys [47,48]. When the cross-sectioned area in the trench was milled further, the existing pores disappeared and other pores appeared. Therefore, it is evidenced that the chimneys are tortuous in nature. It was demonstrated that subcooled nucleate boiling occurred on the cladding tube surfaces under the same thermal hydraulic conditions as used in this work [49]. The morphological feature shown in Figure 6 also provides evidence that the deposits were formed under the subcooled nucleate boiling condition.

The number and size of the pores, i.e., the porosity, seem to increase from the cladding tube surface to the coolant side. Similar trends were observed for the deposit flakes taken from an operating PWR’s steam generator tubes [47]. Small bubbles that have departed from the heated tube surface can coalesce into bigger ones during escape through the tortuous steam chimneys to the bulk coolant, leading to an increase in the porosity of the coolant side. The 2D porosity of the deposits was measured using an image analyzer, and the average porosity was 37% for Case #3 and 39% for Case #4. Therefore, it is concluded that the morphology and porosity of the fuel deposits were not affected by the subsequent exposure to the zinc-containing coolant. On the other hand, as expected from the uneven distribution of the deposits, the thickness of the deposits was not also uniform and thus dependent on the locations, ranging from about 35 to 110 µm.

**Figure 7:**
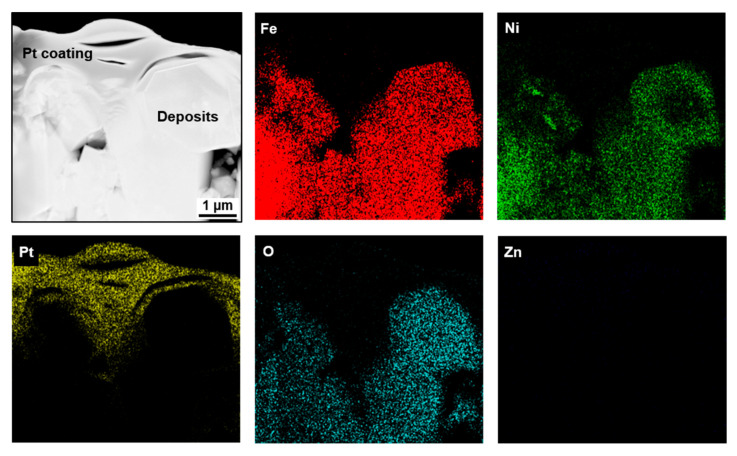
STEM image and EDS elemental mapping of fuel deposits formed under the condition of Case #3.

**Figure 8:**
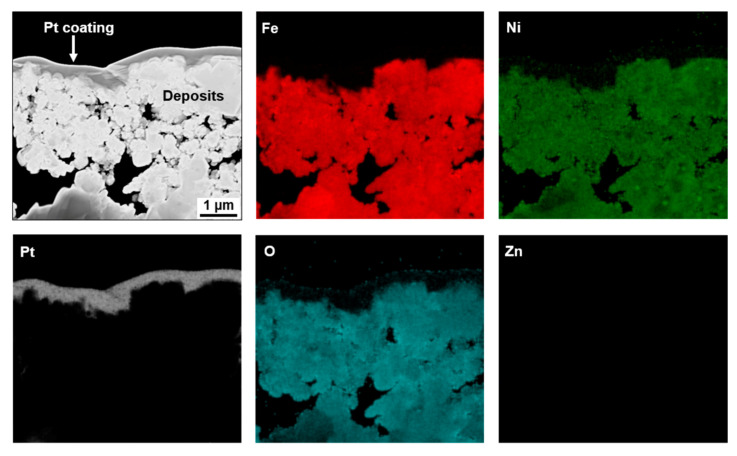
STEM image and EDS elemental mapping of the fuel deposits after the pre-existing deposits were exposed to the 60 ppb of zinc coolant under the condition of Case #4.

To examine zinc-incorporation behavior via the chemical composition of the deposits, TEM specimens were prepared from the deposits using the FIB. The specimens were milled from the coolant side of the deposits because the outer deposits were easily in contact with the flowing coolant containing zinc. Figure 7 presents a STEM micrograph and elemental EDS maps of the fuel-deposit particles formed under the condition of Case #3 without zinc. The EDS maps indicated that the deposits were composed of Fe, Ni, and O. From the STEM-EDS analyses, the deposits were characterized as nickel ferrites with a spinel stoichiometry of Ni_0.45_Fe_2.55_O_4_ on average. These deposits have a large fraction of Ni content compared with the deposits of Case #1. This is because the Case #3 deposits were formed under the condition containing a higher Ni source fraction (Table 2 and Table 3). However, surprisingly, no zinc was detected in the deposits of Case #4, as shown in Figure 8, although the pre-formed deposits were exposed to the 60 ppb of zinc coolant for 500 h. Only a maximum of 0.26 at.% zinc was detected from EDS point analysis. This result was not changed, even after repeating the experiment once more. The chemical composition of the Case #4 deposits was Ni_0.43_Fe_2.57_O_4_ on average, similar to that of Case #3, indicating that neither was zinc incorporated in the deposits nor were nickel and iron in the deposits released into the coolant during the exposure to the zinc-containing coolant. XRD examination was not carried out because the chemical compositions of both the Case #3 and Case #4 deposits were nearly the same.

Zinc acetate dissolves in the primary coolant to become zinc divalent cations and acetic ions. The dissolved Zn^2+^ ions have been reported to replace cations such as Ni^2+^, Co^2+^, and Fe^2+^ in newly forming spinel oxides on the fresh surfaces and in the pre-grown oxides of Ni-based alloys and stainless steels in the RCS coolant environments of PWRs [7,8,9,10]. The substitution of zinc cations in the spinel oxides can be attributed to their higher tetrahedral site preference energy in the oxides, compared with those of other cations [50,51,52]. Zinc incorporation in fuel deposits has also been reported in operating PWRs with zinc water chemistry [29,30,31]. However, in those instances, it was hard to determine whether the zinc was incorporated in newly forming deposits after zinc addition, in pre-existing deposits before zinc addition, or in both. From Figure 4, it is now clearly demonstrated that zinc divalent cations dissolved in the RCS coolant are incorporated in newly forming fuel deposits. This indicates that zinc ions are involved in the nucleation and growth process of the deposits, competing with other cations such as nickel, iron, cobalt, and their radioactive isotopes (^58^Co for Ni, ^55^Fe for Fe, and ^60^Co for Co) to occupy the lattice sites. Because corrosion products are continuously released from the RCS materials and transported in the reactor core, new fuel deposits are nucleated and grown on fuel cladding tubes during the operation of PWRs. At this time, due to the incorporation of zinc cations in the newly forming deposits, surplus nickel, iron, cobalt, and radioactive isotopes defeated by zinc remain in the flowing coolant and thus can be removed from the coolant through ion exchange membranes and filters in the system, thereby contributing to a reduction of radiation fields. It is obvious that the replacement of the radioactive isotopes in the coolant has an immediate effect on dose-rate reduction. Because the above metal cations consisting of the fuel deposits are activated in the core [3] and become the major radioactive source, the replacement of the metal ions by zinc ions also results in a radioactivity decrease.

However, zinc incorporation was not observed in the pre-existing deposits, even after exposure to the simulated RCS coolant including 60 ppb of zinc for up to 500 h. In this case, if we simplify the fuel deposits to nickel ferrite (NiFe_2_O_4_) for the convenience of calculation, the zinc-incorporation reaction may occur as follows.

Zn^2+^ + NiFe_2_O_4_ → ZnFe_2_O_4_ + Ni^2+^ (1)

The Gibbs free energy for reaction (1) under the 330 °C test condition is ΔG_330°C_ = −16.6 kJ/mol, which was calculated using the HSC Chemistry 6 software [53]. This means that nickel substitution by zinc cations is thermodynamically spontaneous. In a similar manner, Ni(ZnFe)O_4_ may be formed through iron replacement by zinc cations. Unfortunately, we could not obtain the Gibbs free energy for this reaction because of the absence of the thermodynamic data for Ni(ZnFe)O_4_ at high temperatures. Nickel ferrite has an inverse spinel structure [42,43,44,45]. To incorporate zinc cations into the pre-formed deposits, metal cations positioned in the spinel lattice sites should first be dissolved out from the sites into the coolant, and then, zinc cations in the coolant enter the resultant vacant sites. Therefore, it is believed that the zinc-incorporation process includes the selective dissolution of metal cations from the lattice sites and solid-state diffusion through the vacancies. It was also reported that the free energy of the formation of zinc-containing spinel oxides on stainless steel was larger than the energies for the zinc substitution of divalent cations such as nickel, iron, and cobalt [11]. That is, the formation of zinc-incorporated spinel is thermodynamically favored. Consequently, it is thought that the rate of zinc substitution into the pre-formed deposits is very slow.

It was reported that the initial addition of zinc to the RCS of operating PWRs led to an almost immediate increase in nickel and radiocobalt activity concentrations in the coolant [34,54]. The increase has been attributed to an exchange of zinc with the nickel and radiocobalts in the oxides already existing on the material surfaces of the primary system. Therefore, based on field experience together with this study, the immediate increase could result from the release of the replaced nickel and radiocobalts from the oxides of Ni-based alloys and stainless steels, not from fuel deposits.

## 4. Conclusions

This paper focuses on the behavior of zinc incorporation into fuel deposits on zirconium-based fuel cladding tubes being newly formed after zinc addition and having already been formed before zinc addition into the simulated RCS coolant at 328 °C. Dissolved zinc cations were incorporated into newly forming fuel deposits, indicating that the replacement of nickel, iron, and their radioactive isotopes by the zinc contributes to a reduction of the radiation fields of PWRs. The zinc incorporation was confirmed by STEM-EDS analyses and resulted in a decrease in the lattice constant of the deposits, which was mainly due to the decrease in iron content with a larger ionic radius in the spinel deposits. However, zinc was not detected in the pre-formed deposits, which were subsequently exposed to the 60 ppb of zinc coolant for 500 h. These results indicate that the energy necessary to replace the metal cations in the spinel lattice sites with zinc cations in the coolant is significantly larger than that to form new zinc-containing spinel deposits.

## Figures and Tables

**Table 1:** Chemistry and mechanical properties of fuel cladding tubes.

Chemistry (wt.%)					Mechanical Property		
Sn	Fe	O	Nb	Zr	Yield Strength (MPa)	Ultimate Tensile Strength (MPa)	Elongation (%)
0.93	0.11	0.12	0.93	Bal.	612.5	819.2	15.8

**Table 2:** Main conditions for the tests for zinc incorporation into newly forming fuel deposits.

	Coolant Chemistry in the Autoclave	Test Duration	Others
Deposition of fuel deposits without zinc (Case #1)	RCS coolant: 1000 ppm B + 2 ppm Li + No Zn Deposit source: 0.5 ppm Fe + 0.28 ppm Ni	600 h	DO < 5 ppb DH: 35 cm^3^/kg H_2_O 328 °C, 130 bar Heat flux: 65 W/cm^2^
Deposition of fuel deposits with zinc (Case #2)	RCS coolant: 1000 ppm B + 2 ppm Li + 20 ppb Zn Deposit source: 0.5 ppm Fe + 0.28 ppm Ni		

**Table 3:** Experimental conditions for the tests for zinc incorporation into pre-existing fuel deposits.

	Coolant Chemistry in the Autoclave	Test Duration	Others
Deposition of pre-existing deposits (Case #3)	RCS coolant: 1000 ppm B + 2 ppm Li + No Zn Deposit source: 8 ppm Fe + 6 ppm Ni	240 h	DO < 5 ppb DH: 35 cm^3^/kg H_2_O 328 °C, 130 bar Heat flux: 65 W/cm^2^
Exposure of the pre-existing deposits to the zinc-containing coolant (Case #4)	RCS coolant: 1000 ppm B + 2 ppm Li + 60 ppb Zn Without deposit source	500 h	
